# Supplementary material for: Depressive symptoms and associated factors among HIV positive patients attending public health facilities of Dessie town: A cross-sectional study
Source: PLoS One. 2021 Aug 5;16(8):e0255824. doi: 10.1371/journal.pone.0255824 (PMC8341501; doi:10.1371/journal.pone.0255824)
Supplement: Attachment — Submitted filename: Response to Reviewers.docx [file pone.0255824.s004.docx]

**Colette Smith, PhD**

**Academic Editor, PLOS ONE**

**RE-2: Manuscript ID:** PONE-D-21-03982R1 (Depression and associated factors among HIV infected patients attending public health facilities of Dessie town: A cross-sectional study.)

**Dear Dr. Colette Smith,**

Thank you very much for your email and the comments/suggestions of the reviewers and academic editor. We have looked at the comments and have revised our paper accordingly. We hope our paper improved as a result of incorporating the reviewers' and academic editor's comments and suggestions.

Please find for your kind consideration the following:

- A rebuttal letter that responds to each point raised by the academic editor and reviewer labeled 'Response to Reviewers'. The point-by-point responses are written in *italic* font style.
- A revised manuscript with track changes labeled 'Revised Manuscript with Track Changes'.
- A revised paper without tracked changes labeled 'Manuscript'

While hoping that these changes would meet with your favorable consideration, we are happy to hear if there are more comments and suggestions. Please do not hesitate to let us know if you have any questions.

Yours Sincerely,

Yitayish Damtie

School of Public Health, Wollo University

Dessie, Ethiopia

Tel:+251943517982

E-mail: [yitutile@gmail.com](mailto:yitutile@gmail.com)

**Point by point response**

Journal Requirements:

Please review your reference list to ensure that it is complete and correct. If you have cited papers that have been retracted, please include the rationale for doing so in the manuscript text, or remove these references and replace them with relevant current references. Any changes to the reference list should be mentioned in the rebuttal letter that accompanies your revised manuscript. If you need to cite a retracted article, indicate the article’s retracted status in the References list and also include a citation and full reference for the retraction notice.

- *Thank you. Reference #1, #18, #24, #29 and #30 have been retracted and replace with relevant current references. But reference #2 is retracted and used as it is due to the absence of relevant literature. We indicated its retracted status in the References list.*

Additional Editor Comments (if provided):

Throughout - please use "HIV positive" or "people living with HIV" or "PLWH" rather than "HIV-infected"

- *Thank you. We have tried to use "HIV positive" throughout the document accordingly.*

Throughout - please refer to "depressive symptoms" instead of "depression".

- *Thank you. The comment is accepted and addressed accordingly.*

Abstract - Please clarify that Dessie town is in Ethiopia and give the calendar dates over which the survey was conducted.

- *Thank you. We have tried to clarify it in the abstract section line 29.*

Abstract - please change line to "PHQ-9) at a cut-off point of 5 was used to assess depressive symptoms". This is because these tools screen for symptomology, rather than confer a clinical diagnosis.

- *Thank you. The comment is accepted and addressed in the abstract section line 33-34.*

Abstract, result: PLease change "The magnitude of depression among..." to "The prevalence of depression among..."

- *We have tried to address it in the abstract section line 39.*

Abstract, result: Please add in what the comparison group was for the age association (e.g. "Age 40-49 compared to 30-39 years (AOR=3.00; "

- *Thank you. The comment is accepted and addressed in the abstract section line 40-41 accordingly.*

Abstract, conclusion: Please change the first sentence to "The prevalence of depression was low compared to previous studies."

- *We have tried to address it in the abstract section line 45 accordingly*

Introduction, line 52: Please reword as "In Ethiopia, it was estimated in 20XX that 399,000 of the urban population were living with HIV/AIDS."

- *Thank you. The comment is accepted and addressed in the introduction section line 55-56 accordingly.*

Introduction, line 56: Please change to "It disproportionately affects people living with HIV, with a recent meta-analysis indicating a two-fold higher risk compared to the general population [8]".

- *Thank you. We have addressed it in the introduction section line 58-59 accordingly*

Introduction, line 58-60 (sentence beginning "The lifetime prevalence of depression..."). I would suggest cutting this sentence as it contradicts the sentence in lines 55-56, and the context for these numbers is not clear.

- *Thank you. We have tried to remove it accordingly.*

Introduction, line 61-62. I would suggest rewording as "In Ethiopia, previous studies assessing the prevalence of depression among PLWH have differed considerably, with estimates ranging from 11.7-76.7% [11-15]."

- *The comment is accepted and addressed in the introduction section line 61-63 accordingly.*

Introduction, line 71. Please change to "Depression has been associated with a wide range of factors, including age, sex ...”.

- *Thank you. We have tried to address it in the introduction section line 72-73 accordingly.*

Introduction, lines 78-81. Is this paragraph necessary? Although i completely agree that ending the HIV/AIDS epidemic is important, its direct link to depression requires more explanation, and is not directly relevant to the manuscript. So this section could be cut.

- *Yes it is necessary. Depression imposed substantial effects on the health of HIV positive patients. It leads to poor ART drug adherence and further transmission of HIV/AIDS. One of the seventeen goal of SDG is to end the HIV/AIDS epidemic by 2030. So, it is difficult to achieve SDG goal (end AIDS epidemic) in the presence of depression as it leads to poor ART drug adherence and further transmission of HIV/AIDS. We have tried to revise it in the introduction section line 81-83.*

Methods, line 88. Please clarify that Dessie town is in Ethiopia, and perhaps indicate where Dessie is within Ethiopia (e.g. "A cross-sectional study was conducted between 1-30 March 2019 in public health facilities of Dessie Town, which is a city located within north-central Ethiopia").

- *Thank you. We have tried to clarify it in the method section line 91-94 accordingly.*

Methods, line 91. Should this be "The study population was randomly selected from all adults with HIV attending public health facilities...."

- *We have accepted the comment and addressed it in the method section line 96-97 accordingly.*

Methods, sample size. Do you mean that you assumed that 10.1% would have depressive symptoms (so that 89.9% did not)? Otherwise this prevalence seems a little high.

- *Thank you. It is editorial issue. It is to mean that 10.1% of patients without ART side effects have depressive symptoms.*

Methods, lines 95-108. This section is perhaps a little long. It could be considerable shortened. For example: "The study sample size was calculated for the primary risk factor as presence of antiretroviral drug side effects, considered as a binary variable. Based on previous research [12], we wished to to detect differences in adjusted odds ratios of at least 4.7, assuming an underlying prevalence of 10.1%, power of 80% and 5% Type I error. After adjustment for 10% non-response, the minimum required sample size was 388. Systematic random sampling was used to select participants using the antiretroviral drug dispensary registration book as the sampling frame. Study participants were invited to participate when attending the health facility for antiretroviral drug collection."

- *Thank you. The comment is accepted and addressed in the method section line 100-107 accordingly.*

Methods, line 111. Please clarify if the card reviewed was the medical record?

- *Yes it was the medical record. We have tried to clarify it in the method section line 110 accordingly.*

Methods, line 114. Please consider rewording as "The questionnaire collected data on socio-demographic, clinical, psycho-social, and behavioral characteristics."

- *We have revised it in the method section line 113-114 accordingly.*

Methods, line 116. Please clarify that the PHQ-9 is used to measure depressive symptoms rather than depression directly. Please also clarify that the cut-off of 5 typically corresponds to mild, moderate or severe depressive symptoms as other cut-offs are sometimes used

- *Thank you. We used PHQ-9 scale to assess depressive symptoms. In the PHQ scale, a score of 0-4, represents no depression and a score of 5-9, 10-14, 15-19 and 20-27 represent mild, moderate, moderately sever and sever depressive symptoms respectively. In this study, PHQ score ≥ 5 was used as a bench mark to categorize depression. Accordingly, patients said to have depressive symptoms if they scored ≥ 5 and have no depressive symptoms if they scored <5.*

Methods, line 125. Please give the median score (out of 10?) used to define "high" and "low" stigma. Also, where was this median obtained from, as it appears that only 30% had perceived stigma in Table 2 (instead of the 50% you would expect if the median was taken from this present study).

- *Stigma was assessed by using ten ‘yes’ or ‘no’ questions and the score ranged from 0 to 10. In our study, out of 380 study participants, 266 of them scored 0, 11 of them scored 1, 4 patients scored 2, 9 patients scored 3, 14 patients scored 4, 29 patients scored 5, 23 patients scored 6, 16 patients scored 7, and the remaining 8 patients scored ≥8 out of 10. The median score is the middle number in a given sequence of numbers when it’s ordered by rank. In this study, the median score is 0. Accordingly, having perceived stigma was defined if patients scored above the median score (i.e. above 0) which is 30%. Perceived stigma cannot be 50% since a score of greater than the median value was used to define perceived stigma.* *You can confirm it in manual calculation using the above frequency distribution. Median value can be calculated manually or using SPSS.*

Methods, line 131. as the study is cross-sectional, the independent variables are not really "predictors". Perhaps "explanatory variables", "factors" or "independent variables" would be better?

- *We have tried to use the term "independent variables" accordingly.*

Methods, line 135. As this is a cross-sectional study, it may not be appropriate to use the word "determinents". "associated factors" could be an alternative?

- *We have tried to use the term "associated factors" accordingly.*

Results, line 152. It would be good to give the overall prevalence of depression (for the whole population), before giving it in the sub-groups.

- *We have tried to put the overall prevalence of depression in the result section line 154 accordingly.*

Results, Table 3. Would it be possible to add the question used to define the variable "HIV sero-status disclosure" in a footnote to the table?

- *We have tried to put it as a table footnote accordingly*.

Results, Table 3. CD4 count, opportunistic infections and WHO stage are quite correlated - is it reasonable to include all in the model?

- *We have tried to re-analyze the data by removing ‘CD4 count’ and ‘WHO stage’ from the model accordingly.*

Discussion, line 227. Please say "times the odds" rather than "times the risk" here.

- *Although it is not clear, we have tried to revise it as “The odds of depressive symptoms were 2.6 times more among patients who took medication other than ART as compared to their counterparts”.*

Discussion, line 237. You could consider adding the word all, so it reads "cannot be generalised to all people living with HIV".

- *The comment is accepted and addressed accordingly.*
